# Structure Modeling of the Norepinephrine Transporter

**DOI:** 10.3390/biom10010102

**Published:** 2020-01-07

**Authors:** Izabella Góral, Kamil Łątka, Marek Bajda

**Affiliations:** Department of Physicochemical Drug Analysis, Faculty of Pharmacy, Jagiellonian University Medical College, Medyczna 9, 30-688 Cracow, Poland; izabella.goral@student.uj.edu.pl (I.G.); kamil1.latka@uj.edu.pl (K.Ł.)

**Keywords:** norepinephrine transporter, homology modeling, ligand docking, reuptake inhibitors

## Abstract

The norepinephrine transporter (NET) is one of the monoamine transporters. Its X-ray crystal structure has not been obtained yet. Inhibitors of human NET (hNET) play a major role in the treatment of many central and peripheral nervous system diseases. In this study, we focused on the spatial structure of a NET constructed by homology modeling on *Drosophila melanogaster* dopamine transporter templates. We further examined molecular construction of primary binding pocket (S1) together with secondary binding site (S2) and extracellular loop 4 (EL4). The next stage involved docking of transporter inhibitors: Reboxetine, duloxetine, desipramine, and other commonly used drugs. The procedure revealed the molecular orientation of residues and disclosed ones that are the most important for ligand binding: Phenylalanine F72, aspartic acid D75, tyrosine Y152, and phenylalanine F317. Aspartic acid D75 plays a key role in recognition of the basic amino group present in monoamine transporter inhibitors and substrates. The study also presents a comparison of hNET models with other related proteins, which could provide new insights into their interaction with therapeutics and aid future development of novel bioactive compounds.

## 1. Introduction

The norepinephrine transporter (NET) belongs to the family of sodium-coupled ion-dependent transporters (SLC6). In high density NET occurs in the plasma membranes of noradrenergic neurons and also in glial cells [1]. It is physiologically responsible for norepinephrine (NE) transport from the synaptic cleft into presynaptic neurons [2]. The family of Na^+^ dependent transporters consists of many proteins and includes i.e., transporters of amino acids, such as leucine transporter (LeuT) and biogenic amine reuptake proteins, such as dopamine (DAT) and serotonin (SERT) transporters. All of these share a resemblance in amino acid sequences, 3D protein structures, and transport mechanisms for their substrates [3]. Transport by all neurotransmitter sodium symporters (NSSs) depends on Na^+^/K^+^ ATPase which maintains the concentration gradient of Na^+^ ions at both sides of the cell membrane [4]. This electrochemical potential is a driving force for translocation of one molecule of the substrate along with sodium and other ions, which results in intracellular accumulation of neurotransmitter [3]. NET and DAT are responsible for transport of each norepinephrine or dopamine molecule with one or two sodium and one chloride ions. The SERT co-transports molecule of serotonin (5-HT) with one Na^+^ and Cl^−^ ions, while in the opposite direction a single potassium K^+^ ion is moved [5].

By regulating the concentration of released norepinephrine in the synaptic cleft, NET protein plays a critical role in many central and peripheral mechanisms, such as cardiovascular effects or behavioral processes. Blockage of NET has proven useful in the treatment of a variety of central nervous system (CNS) disorders, such as attention deficit hyperactivity disorder (ADHD), panic and suicidal disorders, or depression [2]. The activity of NET can be modulated by both biogenic substrates and selective or nonselective inhibitors of monoamine transporters. However, molecular mechanisms involved in the action of this protein are still not fully understood [6]. Crystal structures of the transporters may be useful in understanding the processes which they mediate. The Protein Data Bank provides X-ray structures of the leucine transporter aLeuT (*Aquifex aeolicus* LeuT) and monoamine transporters (MATs): dDAT (*Drosophila* DAT) and hSERT (human SERT) crystallized in 2005, 2013, and 2016 [7,8,9,10]. The human NET (hNET) crystal structure has not been obtained yet. The molecular structure of the norepinephrine transporter is important for understanding the interactions with its ligands and future development of more active and/or selective compounds. Isolated complementary DNA (cDNA), which encodes the human noradrenergic transporter protein, provided the first information about this structure and revealed that hNET comprises 617 amino acid residues [11,12]. The first significant data about the tertiary structure and functioning of proteins from the SLC6 family came from studies on prokaryotic homolog LeuT received from the bacterium *Aquifex aeolicus* [7]. Analysis of its sequence and X-ray crystal structure revealed 20–25% overall homology with others where ~60% homology has been demonstrated for the core region [9]. Experimental data have confirmed that other human MATs consist of 620 (human DAT, hDAT) and 630 (hSERT) residues and also present high conservation of topological domains whereas the main differences can be found at carboxy terminus [12]. Like all of the monoamine transporters, NET also consists of 12 α-helical transmembrane spanning domains (TM) with *N*- and *C*-termini located at the intracellular side. The primary substrate binding region (S1) occurs between TM1, TM3, TM6, and TM8 [13]. Differences between prokaryotic and eukaryotic members are marked in terms of the extensivity of *N*- and *C*-termini and the prolonged extracellular loop 2 (EL2) with a characteristic disulfide bond located between TM3 and TM4. Mammalian equivalents also possess phosphorylation sites and post-translational modifications not present at prokaryotic transporters [14,15]. A series of recombinant chimeric transporters, combining features of NET and DAT, have turned out to be helpful in the identification of particular structure determinants engaged in the ligand binding and substrate transport processes. The highest degree of conservation has been observed at the first three transmembrane domains, which also coincides with other family members of Na^+^/Cl^−^ dependent transporters. Therefore, involvement of these three domains in ion-dependence and uptake process is common at all related transporters. The same observations have been found for TM5 to 8, where the chimeras combining NET and DAT caused loss of ability to transport substrates [11,16]. Differences in pharmacological and kinetic profiles between NET and DAT proteins led to the observation that the appreciable carboxy-terminal region derived from TM9 might be responsible for the specific affinity of substrates and stereoselectivity which has been proposed as typical of NET. Moreover, mutations at TM9 are connected with attenuated surface expression and cause orthostatic intolerance [17]. Some characteristic determinants occurring within TM5–8 have been proposed for high affinity binding of tricyclic antidepressants [16]. Mutagenesis studies within TM2 and intracellular loop 1 (IL1), including GXXXRXG motif, pointed this region as involved in regulating the affinity of substrate and inhibitors [18,19]. Also, histidine residues placed at the terminal part of EL2 are important for nisoxetine binding [20]. Mutations within EL3 have a much greater impact due to the change in the architecture of connected TM5 and TM6, as a consequence of affecting the permeation ability between TM1 and TM6 of noradrenaline and inhibition by desipramine [21]. To get a better view into the structures of the transporter, the mechanism of alternating access and its influence on substrate availability should been mentioned. The general principle of action is to allow for access to the ligand binding site in protein from only one (external or internal) side but never from both at the same time. This process prevents any loss of energy, related to uncontrolled movement of ions. The substrate binds to the outward-faced conformation of the transporter. When the binding site is occupied, the external gate closes and internal gate opens. After release of ions and the coupled neurotransmitter to the intracellular space, the internal gate closes. The last part of this mechanism is connected with facing the transporter outward and then the whole cycle is repeated. At the molecular level, translocation of substrate is related to conformational changes of protein first observed at prokaryotic homolog LeuT [1]. This mechanism differs to a certain extent at mammalian equivalents. Core domains represent coordinated hinge movement. In the first step, TM1b and TM6a with extracellular loop 4 (EL4) bend together and allow for penetration to the interior of the transporter. Differences can be found in the movement of the domains located near the cell interior. In prokaryotic homologs, TM1a and TM6b are kept together around the binding site region at a tilt of 25° [22]. While at MATs, the release of substrate to the cytosol is possible because of the outward directed movement of mainly one core domain, i.e., TM1a, and inward movement of EL4 [4]. The mechanism of alternating access to the binding site of prokaryotic LeuT may be explained by the “rocking bundle” hypothesis [22]. The hNET and hDAT are thought to operate by the following stoichiometry: NE/Na^+^/Cl^−^ 1:1:1 for hNET and DA/Na^+^/Cl^−^ 1:2:1 for hDAT. Both of these result in positive charge transfer during the transport cycle [23]. Binding of the inhibitors with the transporters is strictly dependent on Na^+^ ions [24]. LeuT X-ray structure resolved the positions of amino acids at the binding site responsible for coupling sodium ions and determining their features. Residues located in TM 1b and 6a and 7 formed a Na1 site, which is a supplement of the binding pocket, hence making it accessible for substrates. Binding of the Na^+^ ion at the Na1 site derived movement of the transporter toward outward-face, while the presence of an Na^+^ at the Na2 site caused opposite conformational changes [21]. A second cation binding site could be found closer to the intracellular region between TM 1a and 8. During coordination of Na^+^ ion, amino acids residues stitched two domains together, thereby stabilizing them closed from cytoplasmic site conformation. Transition from outward to occluded protein state was triggered by the coordinated action of a substrate with an active site and sodium ion binding to an Na2 site [25]. This mechanism was also supported by molecular dynamics stimulations which revealed that unbinding of Na^+^ from an Na2 site leads to unbinding of substrate molecule [21]. In comparison to other SLC6 members, LeuT represented Cl^−^ independent transport. A single chloride ion was coupled to the EL2 located 24.7 Å away from the important Na1 site. Absence of an anion site in close relation to the binding pocket might explain why Cl^−^ was not involved in substrate translocation [26]. Positions of sodium ions at 4m48 dDAT crystal structure coincided exactly with both Na sites in LeuT. The chloride ion was located adjacent to the Na1 ion which is consistent with findings from mutational study on chloride-dependent LeuT mutant [25].

Herein, we decided to address some essential questions, regarding to the lack of the X-ray crystal structure of human NET and unknown molecular details underlying the mechanism of action for already approved drugs. We wanted to present and discuss molecular details of the spatial architecture of the hNET, and reveal which amino acid residues are responsible for the binding of inhibitors. In this study, we built spatial NET models, docked selected inhibitors into the binding pocket, and evaluated ligand-receptor interactions.

## 2. Results and Discussion

### 2.1. Model Building and Evaluation

To provide structural information for NET, we constructed three-dimensional models built with amino acid sequence of a human norepinephrine transporter on the basis of templates from related homologous proteins. Of all the members of the SLC6 family, human DAT shows the greatest homology to NET with an amino acid sequence identity of 78%. Moreover, both reveal transport of mutual substrates [16].

**Figure 1:**
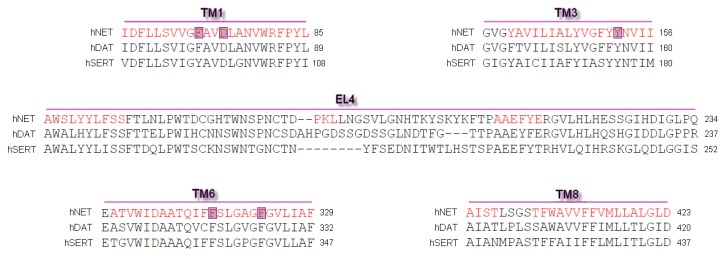
Representation of fragment of sequence alignment for core domains and extracellular loop EL4 of human NET, DAT, and SERT. The most important residues found at hNET models involved in binding ligands are marked in blue. First line depicted as red represented the alpha-helix secondary structure predicted by PROMALS3D [29].

Differences and similarities between human MATs were examined after alignment of protein sequences and their spatial structures. Alignment of sequences was carried out on the PROMALS3D web server [27]. We used sequences of human NET, DAT, and SERT. Primary sequences of mammalian equivalents are strikingly identical, yet their selectivity for substrates and inhibitors included different structural determinants [11,16]. This comparison revealed good alignment of almost all amino acid residues and a high conservation index (Figure 1) [28]. The sequence used for the generation of hNET models represented a deletion of amino acid residues from the polypeptide chain at *N*- and *C*-termini. Residues between A73 and L76 (TM1) and the longer part at TM6 between L319 and L326 were determined hinge regions which possess an unfolded structure.

To create the NET structure we used two of the *Drosophila melanogaster* dopamine transporters (dDAT)—Protein Data Bank (PDB) codes: 4xpg and 4m48—as templates. These two were proposed by a homology-modeling server, which assigned them the highest score, providing further top-rated models. The similarity in sequence between dDAT and human NET reached the level of 63.3%; also, the sequence identity was considerable (49.6%) [30]. Both of the dDAT represented outward-open conformation with a bound cocaine analog and nortriptyline in the central binding site of 4xpg and 4m48 crystal structures, respectively. Inhibitors blocked the transporters from binding with substrate, preventing further conformational changes toward occluded and inward-open state [9,31].

**Figure 2:**
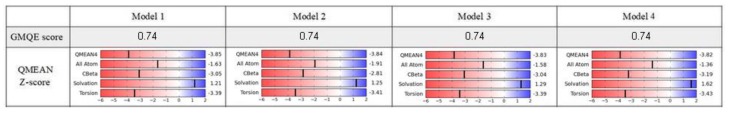
Quality assessment of chosen models.

The homology modeling was carried out on a SWISS-MODEL server [32]. The hNET models were constructed automatically through target-template sequence alignment. Based on the quality assessment, the four top-ranked models were chosen. We took into consideration relevant quality estimations, such as global model quality estimation (GMQE), qualitative model energy analysis (QMEAN), and others (Figure 2).

**Figure 3:**
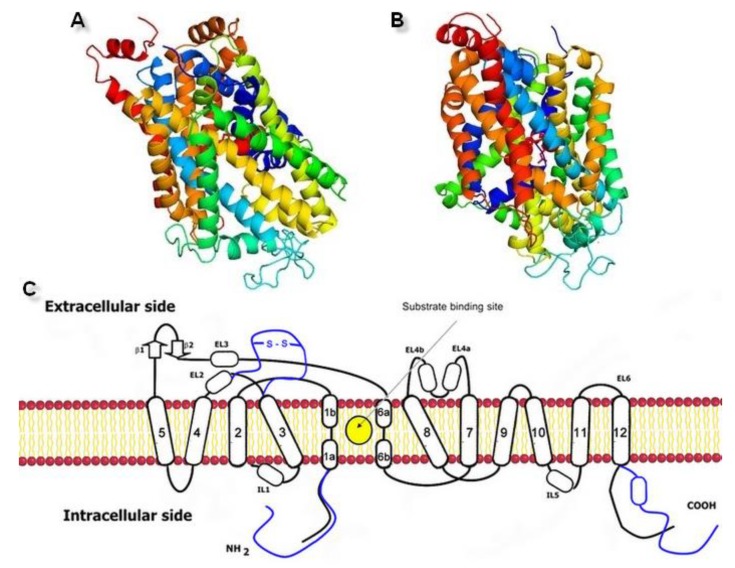
Generated three-dimensional hNET models built on (**A**) 4xpg dDAT template (QMEAN value −3.85) and (**B**) 4m48 dDAT template (QMEAN value −3.82). (**C**) Scheme of the monoamine transporter construction with indicated substrate binding site. Prokaryotic homolog is shown in black, differences found in eukaryotic equivalents in blue.

The GMQE score estimated the quality of each model with different properties resulting from target-template alignment and the method of searching for a template. The score which we obtained rated the represented models with high reliability and accuracy, with values close to 1 rather than 0. Qualitative model energy analysis applied the statistical potential in cases of the comparison of tested models to the experimental structures available in the SWISS-MODEL server database. The QMEAN Z-score parameters evaluated the grade of nativeness of the structural model data on a global scale. QMEAN4 values, which were in closer relation to 0, characterized good agreement of the generated model structures with similar sized experimental structures [33]. All of the results generated for models 1–4 represented a similar range of values from −3.85 to −3.82. In consideration of the obtained results, we used two top-rated and structurally the most different hNET models for further evaluation and docking studies (Figure 3A,B).

**Figure 4:**
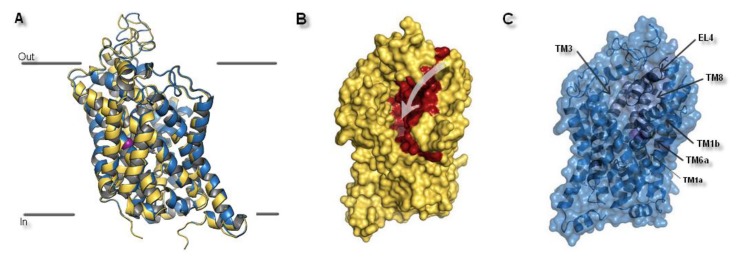
The hNET model presentation: (**A**) Superposition of two selected models. Comparison of the whole proteins. Model built on 4xpg template is shown as yellow, while on 4m48 as blue. Sodium ions are indicated in purple, chloride ions in green. (**B**) Surface of the model built on 4xpg template structure. By visualization of the area occupied by amino acid residues, an access gate to the internal part of the transporter could be easily detected. Surface of core domains and EL4 have been colored in red for contrast. Proposed entry pathway from extracellular site for NET inhibitors are indicated by white arrow. (**C**) Representation of model built on 4m48 structure both as surface and cartoon in blue. Core domains and EL4 are marked in black.

The spatial structure of SCL6 transporters was based on a helical 5 + 5 scheme, where TM1–5 and TM6–10 formed two antiparallel pentahelical clusters aligned to one another. The type of symmetry was a representation of a pseudo two-fold axis arrangement [14]. Based on superposition, it could be ascertained that two selected models were very similar. Small differences between both hNET models could be found in the spatial arrangement of extracellular loops. (Figure 4A). After visualization of the transporter surfaces, a small crevice which led from the external to the interior environments, could be identified (Figure 4B). That fissure provided a pathway by which the hydration of the active site ensued and substrates or inhibitors could reach the high affinity binding pocket.

**Figure 5:**
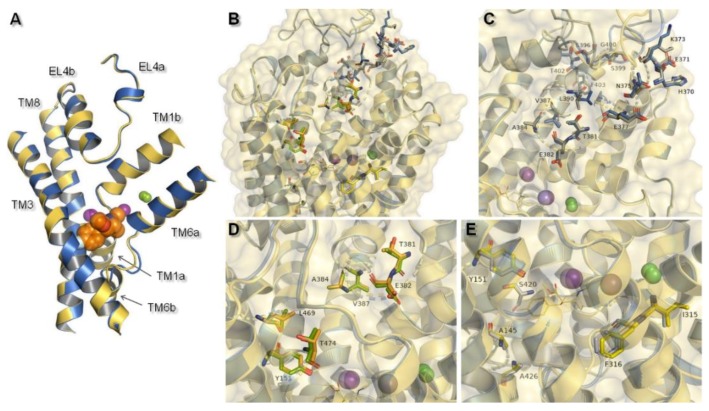
Structural features of hNET models which might be connected with specific interactions with ligands: (**A**) Alignment of core domains and EL4, (**B**) overview of nonconserved residues found between hNET and hDAT represented on received NET models: 4xpg and 4m48. Nonconserved residues are shown for: (**C**) EL4, (**D**) putative secondary binding site, (**E**) primary binding site. For 4xpg, sites are marked in gray, orange, and yellow in (**C**–**E**) respectively, while for 4m48 corresponding ones are shown as blue, green, and gray.

Despite the transient nature of interaction between the external entry pathway and the transported molecule, composition of this extracellular part favored selectivity of NET inhibitors. This assumption was further confirmed by the reported loss of uptake and binding ability, as a consequence of single DAT residue introduction into EL4. As a gatekeeping part, EL4 was highlighted as the first element in recognition of inhibitors and selectivity by the transporter. However, compared to the high affinity binding site (S1), EL4 played only a minor role in substrate recognition [34]. Built models of hNET were consistent with the literature data about monoamine transporters. Superimposition of the transporter core: TM1 and TM3 the bundle and TM6 and TM8 the scaffold domains, together with EL4, which is divided into two subunits (A and B) provided information about general structure of the hNET models. Core domains represented similar configuration. Some distinction could be observed in terms of the connection of subunits of TM6 and the placement in active sites of ions. Also, EL4 differed in its observed secondary structures found at the part of the sequence included at subunit 4a. Glutamic acid E377 and aspartic acid D378 residues presented different conformations. In the 4m48-based model, this fragment was represented as an alpha-helix, while in the 4xpg-based model it created a disordered secondary structure (Figure 5A).

MATs and amino acid transporters were thought to have a secondary binding site (S2), located closer to the extracellular region between EL4 and S1, which was thought to have lower affinity in binding molecules [13]. That additional binding pocket was functionally responsible for allosterically triggering conformational changes from occluded state to inward-facing, and thereby release of ions and associated molecule. This feature occurred as a result of hydration of the primary site [13]. At 4xpg and 4m48, hNET models entry pathway was formed by transmembrane domains TM1, TM3, TM6, TM10, TM11, and extracellular loops EL4 and EL6. Amino acid residues localized in these components of the transporter may take part in binding molecules before they reach S1 site. Such an assumption was confirmed by computational methods with NET models built on LeuT matrix [35]. In the prokaryotic homolog LeuT, precise structural features involved in coupling substrates at site S2 were nondetermined. Despite the lack of information about the exact molecular determinants, several X-ray crystal structures of LeuT presented occupancy by some tricyclic antidepressants (TCAs) and selective serotonin reuptake inhibitors (SSRIs) in this site [22,36]. These findings were supported by experimental data showing an impact of mutations within S1 and S2 on recognition of certain inhibitors by the protein [14]. Changes of a few residues located in the S2 region mostly entailed reduction in the affinity and potency of several inhibitors [37]. Moreover, the halogen binding pocket which has been reported to occur between S1 and S2 was particularly reliable for determining specificity [38]. Other strong evidence of the importance of the vestibular binding site was supported by the fact that binding of the substrate molecule to S2 in the LeuT crystal structure triggered release (in this case of leucine) to the cytoplasm from S1 [37]. Based on molecular dynamics stimulations and mutagenesis studies, the most important residues at the hDAT were detected. First, aspartic acid D79 (corresponding D75 at hNET) residue was indicated, in view of both substrates and inhibitors amine group recognition. The second residue to be indicated was aspartic acid D476 (D473 hNET) as it interacts with hydroxyl moieties [39]. Moreover, studies, carried out on mutated dDAT containing nonconserved hNET and hDAT residues within the substrate/inhibitor pathway to the intracellular space, supported the idea that these residues control selectivity at hNET but not at hDAT. As key determinants, amino acids within S1 were indicated [34]. Due to the lack of clear demarcation between vestibular and primary binding pocket several residues belonged to both S1 and S2. This statement could be confirmed by the fact that two binding sites overlapped also at hSERT [40]. At 4xpg and 4m48, hNET models centrally localized tyrosine Y151 and Y152 could be equally a component both of S2 and S1. As a nonconserved, residues should be highlighted tyrosine Y151 from TM3, threonine T381, glutamic acid E382, alanine A384 and valine V387 from EL4, and leucine L469 and threonine T474 from TM10 at the putative secondary binding site (Figure 5D). At the primary binding site, nonconserved amino acids were alanine A145 and tyrosine Y151 from TM3, isoleucine I315 and phenylalanine F316 from TM6, and serine S420 and alanine A426 localized at TM8 (Figure 5E).

**Figure 6:**
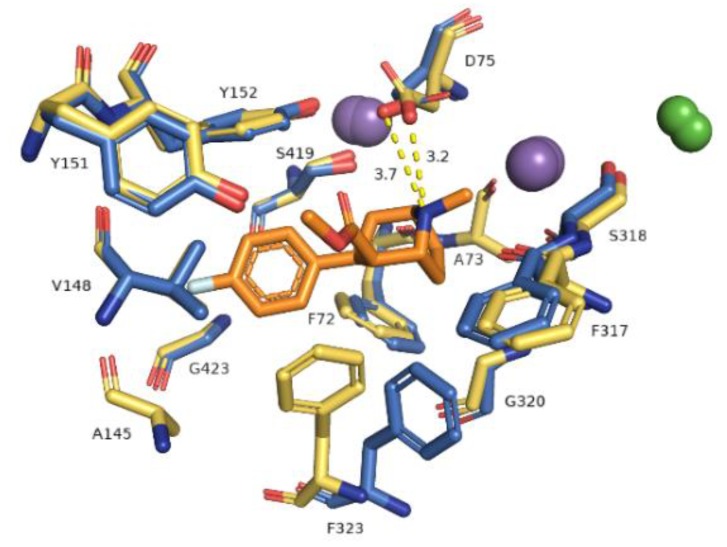
Representation of primary binding site at generated hNET models within 4 Å of the ligand. Residues are colored according to the models from which they originate, i.e., 4xpg model as yellow sticks, 4m48 model as blue. Sodium ions are shown as purple spheres, chloride ions as green. Identified ionic interactions are represented as yellow dashed lines.

The primary binding site in our hNET transporter models appeared approximately midway across the membrane bilayer and for 4xpg hNET model presented occupancy by a small molecule: methyl (1*R*,2*S*,3*S*,5*S*)-3-(4-fluorophenyl)-8-methyl-8-azabicyclo[3.2.1]octane-2-carboxylate. The compound was inherited from the dDAT template (4xpg). The aforementioned cocaine derivative was active against MATs including hNET with the value K*_i_* = 635 nM (pK*_i_* = 6.2) [41]. The compositions of binding pockets in hNET were responsible for high selectivity and affinity in ligand recognition [34]. For assessment of possible interaction between retained cocaine analog and models, we conducted superimposition of protein binding pockets and identified available contacts within 5.5 Å of the ligand (Figure 6). If we took into consideration the physicochemical character of amino acid residues arranged at the centrally located binding region, they could be divided into different segments. The binding pocket consisted of a hydrophobic region responsible for coordination of aromatic rings and a hydrophilic site where the aspartic acid D75 residue was situated. The hydrophobic site included residues of alanine A145, valine V148, glycine G149, and tyrosine Y151 and Y152 from TM3, serine S419 and S420, glycine G423, methionine M424 from TM8, and glycine G320 and phenylalanine F323 from TM6. The farther located residues were aspartic acid D473, alanine 477, and isoleucine from TM10. On the other hand, the hydrophilic site consisted of phenylalanine F72 and aspartic acid D75 from TM1 and phenylalanine F317, and serine S318 from TM6. Situated above 4 Å, were valine V74, alanine A77 from TM1 and phenylalanine F316, leucine L319, and valine V325 from TM6. The importance of aspartate 75 residue in forming polar contacts should be emphasized. Mutation studies revealed that replacement of this residue with alanine, glycine, or arginine caused a loss of transport ability [12]. Measurements of distance between nitrogen atom from the azabicyclooctane ring and oxygen atoms from side chain carboxyl of D75 revealed a close arrangement of 3.2 Å in 4xpg model, while in 4m48 model the distance was 0.5 Å longer. Other residues which could be involved in creating bonds were tyrosine Y151 and Y152 phenyl rings seizing the ligand molecule from the external part of TM3. Phenylalanine F72 and F317 provide binding from the opposite site. F323 residue from TM6 was spatially differently arranged and that fact may have an impact on forming interaction with ligands. Aromatic moieties of F323 were located 3.4 Å apart from each other, measured from the center of rings. The inherence of identified amino acid residues overlapped with several so far created experimental mutants of homologous proteins containing the amino acid sequence of hNET at the binding site [20,42]. Comparison between hNET models and their dDAT templates revealed small differences. At 4xpg model, the hNET side chain carboxyl group D75 (D46 at dDAT) was pointed toward center of the binding pocket, while the *Drosophila* DAT one was bent closer to the Na^+^1 ion with a shift of 0.8 Å. At 4m48, the hNET model and 4m48 X-ray dDAT, this difference was half the size (0.4 Å). The composition of residues within direct range of 4 Å from ligands also differed a little. At dDAT crystal structure (4xpg) with a cocaine analog, a second tyrosine Y123 (Y151 at hNET model) residue from TM3 could be observed. The 4m48 hNET model superimposed to 4m48 dDAT with TCA presented additional glycine G149, serine S420, and alanine A477. All of the mentioned residues could be located at both hNET models but had different impact in binding of molecules. G149 (at hNET) was nonconserved in the eukaryotic dDAT homolog, and D121 takes its place.

**Figure 7:**
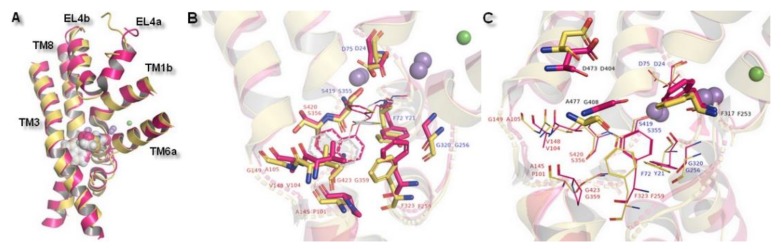
Superposition of hNET model built on 4xpg template presented in yellow and LeuBAT 4mmd shown in magenta. Comparison of (**A**) core domains and extracellular loop 4, (**B**) binding pocket with subsite A and B, (**C**) amino acid residues in subsite C (shown as sticks) together with residues from subsites A&B (shown as lines).

The importance of the results could be supported by a superimposition with another X-ray protein structure which was designed to reflect a biogenic amine transporter: Modified leucine transporter (LeuBAT) from PDB encoded, 4mmd and 4mme (Figure 7). LeuBAT has been constructed as a mutant of wild-type prokaryotic homolog LeuT to provide structural features and pharmacological properties as a hybrid of human BATs. Amino acid residues within active site S1 of LeuT were replaced by the ones from the hSERT sequence. Transporters were isolated from *Aquifex aeolicus* and represented a complex with (S)-duloxetine (4mmd) and mazindol (4mme) molecules. The LeuBAT binding site was divided into specific subsites. Comparison of the LeuBAT binding pocket with the one of the received hNET models may provide subsequent inferences regarding any impact exerted on interaction with ligands. Subsite A formed by residues from TM1, 6, and 8 contained sites where ions bind. Situated at TM1b, D24 was reported to bind compounds by a salt bridge and this corresponded to one in hNET which was also a residue of aspartic acid (D75). Side chain carboxyl group was directed toward the intracellular part of the pocket, while the same atom from D24 (4mmd) was pointed in an external direction to TM1b. The distance measured between carboxyl groups was 3.0 Å. The rest of the corresponding amino acid residues at subsite A possessed a hydrophilic character (from a region closer to TM8) or hydrophobic (at the opposite direction: Phenylalanine F317 and F323, glycine G320). The composition of distal located subsite B presented a sizable niche constructed by nonpolar residues. All of these presented an arrangement of the side chains directed to the interior of the binding pocket. Here, we imply that residues valine V148 and glycine G149 should be identified as initial structural determinants amenable to binding of the compounds in hNET. However, the LeuBAT transporter possessed high affinity only in binding the compounds. Transport activity was not observed and this may affect the reliability of the results [43].

**Figure 8:**
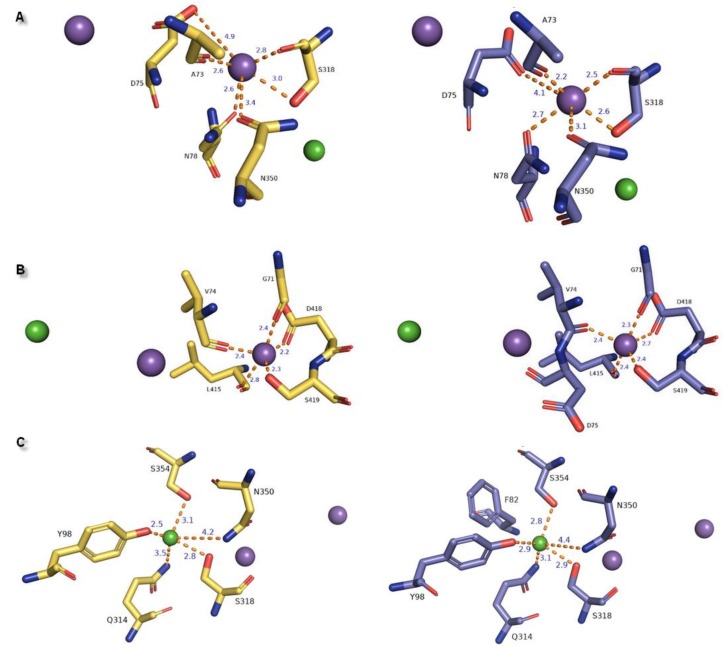
Representation of ion binding sites from different perspectives in generated models: The 4xpg shown in yellow and 4m48 in blue within 4 Å of the ions. Bond length between residues and ion molecules represented as orange dashed lines measured for (**A**) Na^+^1, (**B**) Na^+^2, (**C**) Cl^−^ are shown as purple and green spheres: A/B and C.

In the hNET models built on matrixes 4xpg and 4m48, we observed one chloride and two sodium ion binding sites within 8.0 Å of the ligand inherited from 4xpg dDAT template (methyl (1*R*,2*S*,3*S*,5*S*)-3-(4-fluorophenyl)-8-methyl-8-azabicyclo[3.2.1]octane-2-carboxylate) at regions of TM1, TM6, and TM8 (Figure 8). Two Na^+^ ions were situated at a distance of 7.2 Å and 7.8 Å from each other in 4m48 and 4xpg hNET models, respectively. A chloride ion was found to be the farthest from the ligand in the region of TM6a. Measured distances between chloride and sodium ions were 5 Å (4m48 hNET) and 5.4 Å (4xpg hNET) from central Na1 and 11.8 Å (4m48 hNET) and 12.6 Å (4xpg hNET) from peripheral Na2. To define residues at the binding sites, we analyzed amino acids located 5.5 Å away from each ion. In 4xpg model, the Na1 site consisted of alanine A73, aspartic acid D75, and asparagine N78 residues from TM1b; serine S318 from TM6a; and asparagine N350 from TM7. Na^+^ was coordinated with similar to octahedral geometry derived from the main chain carbonyl oxygen atoms and side chain oxygens. In the 4m48 model, D75 could participate in Na^+^ coordination by a side chain carbonyl oxygen atom which differed from the sodium binding presented at the 4xpg hNET model. In this case, the sodium ion also had a common octahedral coordination geometry, as was observed for prokaryotic homologs [10,39]. At the greater distance within the Na1 site (over 4 Å) valine V74, alanine A77, phenylalanine F317, and leucine L319 residues were situated in both of the hNET models. The Na2 site located closer to TM8 showing trigonal bipyramidal geometry was created through the main chain oxygen atoms of glycine G71 (TM1a), valine V74 from the TM1 hinge region loop, leucine L415 from TM8, and side chains of aspartic acid D418 and serine S419 from TM8 in both hNET models. Oxygen atoms from D75 did not participate in sodium coordination due to their measured distances of over 5.5 Å from the Na^+^2 ion. Other residues within a radius of 5.5 Å were phenylalanine F72, alanine A73, tyrosine Y152, alanine A414, glycine G416, leucine L417, serine S420 at the 4xpg hNET model, and additional valine V70 at the 4m48 hNET model with the lack of serine S420. The anion binding site contained S318 residue also present at the Na1 site, and which was able to interact with both ions by a hydroxyl group. Tetragonal pyramidal coordination of the Cl^−^ was possible due to interplay with oxygen from tyrosine Y98 (TM2), nitrogen atoms from glutamine Q314 (TM6), asparagine N350 amide nitrogen atom, and serine S354 located at the TM7 region. The main chain amide nitrogen atom from phenylalanine F82 was not involved in coupling the Cl^−^ ion, because of the too-large distance separating them in the 4m48 hNET structure. Residues located adjacent to Cl^−^, which did not directly coordinate the chloride anion, are asparagine N78, phenylalanine F82, isoleucine I315, and cysteine C351 at the 4xpg hNET model, and alanine A77, asparagine N78, and cysteine C351 at the 4m48 hNET model. The measured distance between the Na^+^2 and Cl^−^ ions was located at the extreme distance to 12.6 Å in 4xpg and 11.8 Å in the 4m48 hNET models.

General differences in the composition of ion binding sites between the LeuT amino acid transporter and MATs were directly related to ion dependencies. Substitution of Glu290 in LeuT for serine in MATs at the chloride binding region induced a Cl^−^ dependent transport cycle [23]. Residues of amino acids located at the active site were highly conserved in MATs. Received outcomes presented identical amino acid compositions responsible for the ion binding scheme in hNET models corresponding to experimental data (Table 1) [44].

### 2.2. Norepinephrine Transporter Inhibitors

**Figure 9:**
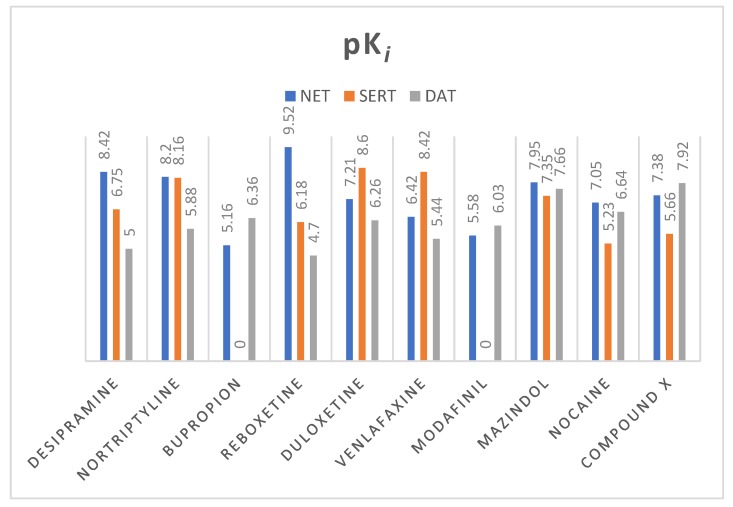
Values of pK*_i_* (negative logarithm of the dissociation constant of ligand—transporter complex) of selected monoamine transporter inhibitors; pK*_i_* have been measured for human MATs. Exceptions are modafinil and compound X whose affinity has been appointed for *Rattus norvegicus* equivalent. All of the values were taken from the ChEMBL Database [46].

From various well-known norepinephrine reuptake inhibitors, 9 have been chosen based on their affinity to human NET and selectivity (Figure 9). The 10th additional compound was a piperidine-based hybrid of nocaine and modafinil as a highly potent MATs inhibitor, referred to subsequently in this paper as “compound X”: 2-({[4-(4-chlorophenyl)-1-methyl-3-piperidinyl]methyl}sulfinyl)ethanol [45].

All of the selected inhibitors may be useful in the treatment of a number of central nervous system (CNS) diseases in which dysfunction of the noradrenergic system is involved. Of the thymoleptic drugs which represent the effect via NET, the following pharmacological classes may be distinguished: tricyclic antidepressants (TCAs), norepinephrine reuptake inhibitors (NRIs), norepinephrine/dopamine reuptake inhibitors (NDRIs), and serotonin/norepinephrine reuptake inhibitors (SNRIs). Stimulation of CNS caused by mazindol, modafinil, and nocaine was reported as a result of NET/DAT inhibition. Mazindol suppresses appetite; therefore, it has been used in the short-term treatment of adiposity. Modafinil exerts wake-promoting activity and thus is prescribed in sleep disorders such as insomnia or hypersomnia. The last one, nocaine, was developed as a substitute agent for cocaine and tested for treatment of addiction in clinical trials [6]. Compound X has been developed as a hybrid of nocaine and modafinil molecules to improve potency toward MATs, but especially for NET and DAT inhibition [45].

**Figure 10:**
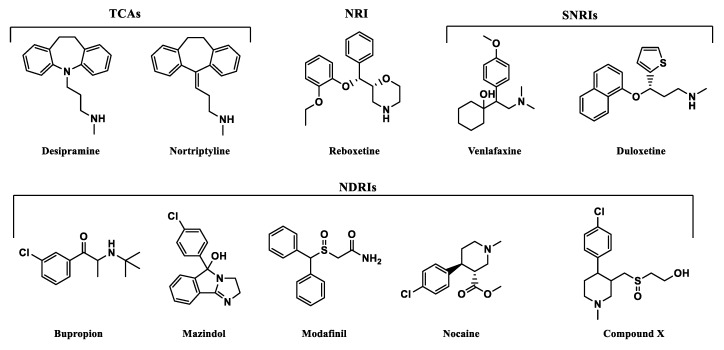
Structures of selected monoamine transporter inhibitors with a representation of pharmacological effects.

Selected compounds presented high diversity in the chemical structures (Figure 10). Both desipramine and nortriptyline share similar tricyclic structures with coplanar architecture. On the other hand, mazindol’s tricyclic ring system built with isoindole and imidazole represents a flat arrangement. The remaining structures contain several separate aromatic and/or heterocyclic rings, or only one, like in bupropion. However, most importantly, they represent the same motif responsible for binding to monoamine transporters (MATs). The relationship between structure and exerted activity has been thoroughly studied and reported elsewhere. Here, it should be mentioned that aromatic residues in close relation to each other and basic amino groups are located 3 to 5 atoms away [47]. Higher affinity to NET was observed for secondary amines, while tertiary ones were more selective for SERT. The type of substitution on the phenyl ring also played a significant role in affinity and selectivity. The presence of an electron withdrawing group (like halogen, haloalkyl, sulfonyl group) affects the extended activity profile also toward SERT or DAT inhibition [47,48]. The substitution of the halogen atom and additional groups at the phenoxy ring are known to be key features that make NRIs specific to NET versus those that make other inhibitors specific to the rest of the monoamine transporters. Single substitution at the second position by a methyl (-CH_3_) or methoxyl group (-OCH_3_) yields norepinephrine specific inhibitors such as reboxetine. Unlike the presence of one or both halogen, methyl or methoxyl groups at the fourth position are responsible for specificity toward SERT [39]. That specific structural insight may have an impact on the recognition of these drugs by NE transporters and placement of the molecules in the binding pocket.

### 2.3. Docking Studies

All of the selected compounds were docked into the binding pocket of selected NET models. Calculated docking scores show that duloxetine, mazindol, nocaine, and compound X fit better to the model built on the 4xpg dDAT template, while the others are better fitted into the 4m48 hNET model (Table 2).

Residues involved in binding were determined and results were confirmed by comparison with literature data [2]. In the 4xpg and 4m48 models of hNET, five residues were involved in creating interactions with compounds: Internally located phenylalanine F72 from TM1b, aspartic acid D75 at the opposite direction from TM1a, tyrosine Y152 from TM3, and phenylalanine F317, F323 from cytoplasmic subunit of TM6. The binding mode of hNET inhibitors could generally be defined by the ionic bond derived from side chain oxygen atoms of D75 and protonated amine moiety of ligands, conformationally different types of π-π stacking created between phenyl rings of Y152, F323, and aromatic residues of each compound. Additional interactions are provided by carboxyl oxygens from F72 and F317 by H-bonds or π-cations derived from the aromatic ring of F72 with protonated amine moieties in NET inhibitors. Aromatic moieties of docked inhibitors are surrounded by hydrophobic residues located closer to the secondary binding site.

**Figure 11:**
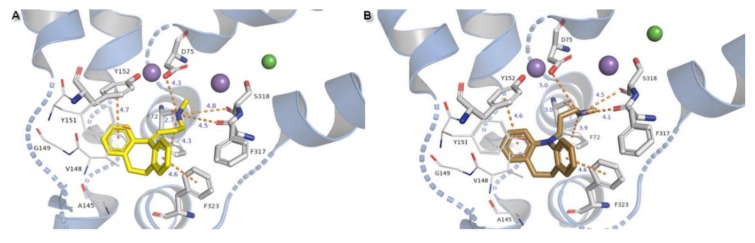
Binding mode of TCAs (**A**) nortriptyline, (**B**) desipramine in hNET on 4m48 template. Initial contacts with measured distances between inhibitors and transporter are shown as orange dashed lines. Amino acid residues involved in binding drug molecules have been represented as sticks. The remaining residues also important in recognition are shown as lines. Core domains are represented as a blue cartoon, the other transmembrane spanning domains are omitted for clarity.

TCAs presented the same arrangement of the tricyclic moiety in the active site of the NE transporter. Both aromatic rings form parallel-displaced π stacking with Y152 and F323 with a bond length of 4.6 Å for desipramine and 4.6–4.7 Å for nortriptyline and remain in close contact with valine V148 which is important in recognition at hNET. The basic amino nitrogen atom is coordinated by main chain carbonyl oxygen atoms from residues F72, F317, and also with serine S318 by H-bonds. Change of the nitrogen at the tricyclic ring system into a carbon atom with sp2 configuration in the nortriptyline molecule has an impact on placement of the propylamine chain. In this case, the salt bridge formed with D75 is shorter in length and would be stronger than that observed for desipramine. The protonated nitrogen atom could be coordinated by a π-cation from F72 with a bond length of 4.3 Å for nortriptyline and 3.9 Å for desipramine (Figure 11).

**Figure 12:**
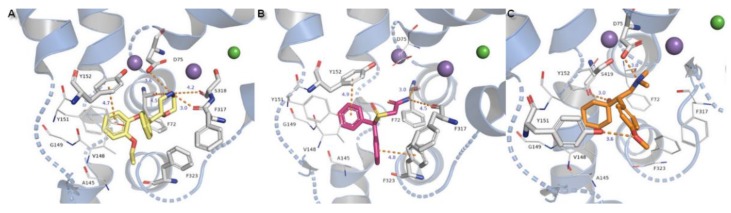
Binding mode of (**A**) reboxetine, (**B**) modafinil, (**C**) venlafaxine in hNET on 4m48 template. Initial contacts with measured distances between inhibitors and transporter are shown as orange dashed lines. Amino acid residues involved in binding drug molecules are represented as sticks. The remaining residues also important in recognition are shown as lines. Core domains are represented as a blue cartoon, the other transmembrane spanning domains are omitted for clarity.

Binding of selective inhibitor reboxetine to the S1 site has been proposed as the coordination of an amine group from the heterocyclic morpholine ring with D75 by a strong ionic bond with a length of 3.6 Å and in addition also with residues of F72, F317, and S318. The lengths of the H-bonds formed by residues from subunit TM6b are likewise shorter for this inhibitor molecule which could elucidate the selectivity of inhibition by that drug to hNET. Only one phenyl ring could be stabilized in the active pocket by T-shape π-π interaction derived from Y152. A second aromatic reboxetine moiety is separated by a greater distance from Y152 (5.8 Å) and F323 (6.0 Å) (Figure 12A).

The modafinil molecule could interact via a nitrogen atom from the amide with the F72 side chain aromatic ring. Typical ionic bond with D75 is not detected due to the presence of amide nitrogen non-ionized in physiological pH. Except for the mentioned difference, modafinil is typically kept in the binding site by hydrophobic residues from TM3 and also forms H-bonds with main chain oxygen atoms from F72 (162.6°) and F317 (162.5°) (Figure 12B).

Binding of the venlafaxine is formed by interplay between the amine moiety and D75 by a salt bridge of 3.0 Å length and angle of 164.1°. The phenol group from the side chain of Y151 and oxygen from the methoxyl moiety create an H-bond with a distance of 3.6 Å. The not observed earlier S419 derived H-bond is 3.5 Å in length and angle 135.1°. The π-π stacking with Y152 or F323 could not be detected due to the different arrangement of the aromatic venlafaxine moiety and distance from the mentioned residues (Figure 12C).

**Figure 13:**
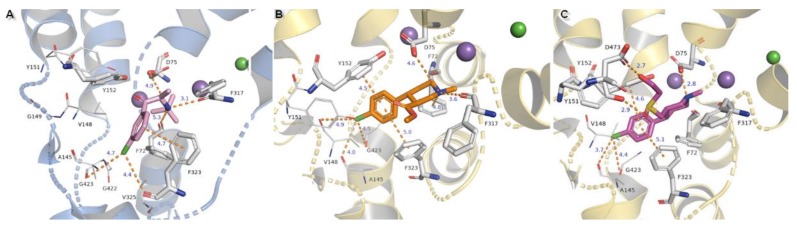
Binding mode of (**A**) bupropion in 4m48 model, (**B**) nocaine, (**C**) compound X in 4xpg hNET model. Amino acid residues involved in binding drug molecules are represented as sticks. The remaining residues also important in recognition are shown as lines. Core domains are represented as a blue cartoon, the other transmembrane spanning domains are omitted for clarity.

Compounds substituted by a halogen atom at the aromatic ring represent insertion of a chloride atom into the niche where hydrophobic residues are located (Figure 13). For bupropion, halogen bond could be derived from the main chain oxygen atom from glycine G423 (4.7 Å) and F323 (4.4 Å). Chlorine moiety from nocaine is bound by G423 and by additional interaction with V148 and alanine A145. Docking of bupropion molecules presents polar contacts with D75. Another interaction is created by F72 aromatic residue which bound to the inhibitor’s amino group by π-cation. The π-π stacking provided from F323 is observed here in a T-shape position. Detection for nocaine T-shaped π-π stacking is provided by both aromatic rings from exterior and interior sides. The basic nitrogen atom from the piperidine ring of compound X remained in closed arrangement to D75 with by far the strongest salt bridge of all of the halogen substituted agents. A hydroxyl group penetrated to the exterior side of the pocket and created an H-bond (2.7 Å, 163.4°) with a side chain carbonyl from D473 located at TM10. Aromatic rings are typically bound with Y152 and F323 which are close to T-shape position of π-stacking. Oxygen from a sulfinyl group could also be coordinated by an H-bond derived from a phenyl group of Y151 (2.9 Å, 137°). Protonated amine moieties from all these three compounds could not be stabilized by π-cations of aromatic F72 rings due to the extreme distances between them.

**Figure 14:**
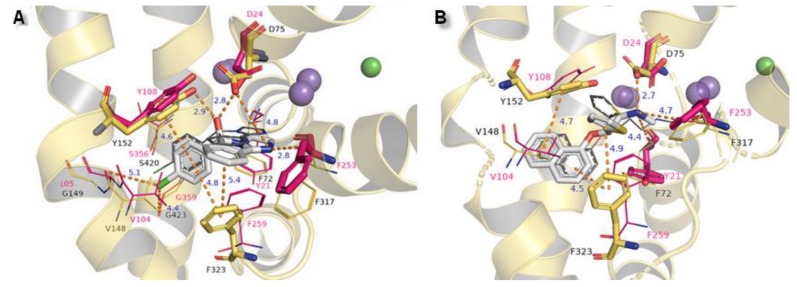
Comparison of the binding mode of (**A**) mazindol, (**B**) duloxetine at superimposed protein structures of hNET on 4xpg template and LeuBAT crystal structures: (**A**) 4mme, (**B**) 4mmd. Amino acid residues are colored according to the proteins from which they originate: 4xpg as yellow and 4mme, 4mmd as pink. Duloxetine and mazindol molecules docked to hNET are presented as light gray sticks, while inhibitors crystallized with LeuBAT are dark gray lines. The most important residues involved in binding drug molecules are presented as sticks. Measured distances between particular functional groups and docked compounds in 4xpg hNET are shown as orange dashed lines, while in 4mme and 4mmd they are omitted for clarity. Sodium ions are shown as purple spheres, chloride ions as green for hNET and LeuBAT alike.

The binding modes of two other NET inhibitors presented below may provide evidence about the validity and rightness of received hNET models and placement of docked molecules in the binding pocket. Amino acid residues, especially those involved in forming contacts, presented close arrangement to aligned proteins of LeuBAT, PDB 4mmd, and 4mme described in model evaluations. Mazindol surrounded by hydrophobic residues of F72, V148, Y152, and F323 (at 4mme Y21, V104, Y108, and F259, respectively) held the inhibitor at the active site of hNET model in a similar way to that observed for LeuBAT. The basic amino group from the imidazole ring is coordinated by an ionic bond from D75 with a distance of 4.8 Å. The H-bond between the protonated amine group from the imidazole ring and the carbonyl group of F317 is observed (2.8 Å). The hydroxyl group interacts by H-bonds with side chain oxygens of D75 (2.8 Å) and a phenol group of Y152 (2.9 Å). The chlorophenyl ring interacted with the Y152 aromatic ring located at a distance of 4.6 Å. A halogen atom is inserted in the site formed by G149, S420, and G423 where glycine G149 at 4xpg is nonconserved at 4mme; the corresponding amino acid is alanine A105. Halogen bonds could be formed between chloride atoms and by main chain carbonyl oxygen atoms of V148 and stronger ones by G423 (distance of 4.4 Å). These interactions could be marked as being particularly important, which is supported by mutagenesis study [8]. Additional, but weaker than that observed for Y152, π-π stacking may be created by F323 with chlorophenyl and isoindole mazindole rings (Figure 14A). The SNRI duloxetine binds to the primary site in an analogical way to that seen in almost all the docked inhibitor structures presented above. The amine moiety is coordinated by the strongest salt bridge from D75 so far, with a measured distance of 2.7 Å, and also by carbonyl atoms from F72 and F317. Despite the similarities in the spatial arrangement of aromatic rings from duloxetine docked to the 4xpg hNET model and 4mmd crystal structure in the binding pocket, the aliphatic chain with a secondary amine moiety in 4xpg is bent in the opposite direction to that observed in 4mmd. This fact has an impact on the strength and type of generated interactions. The salt bridge between the duloxetine nitrogene atom and D24 at 4mmd is created by carboxyl group and located 2.6 Å far. Aromatic naphthalene and thiophene rings are typically bound to Y152 from extracellular site and by F323 from intracellular site (Figure 14B).

To summarize the spatial arrangement of docked inhibitors at the primary binding region, several important observations should be noted. Firstly, the nitrogen atom from the amine moiety remains in close contact with residues of phenylalanine F72, aspartic acid D75 from core domain 1, and amino acids from TM6-phenylalanine F317 and serine S318. All docked inhibitors are bound by protonated amine moieties to D75 with ionic bond, with one exception of modafinil. Aromatic rings are stabilized by hydrophobic π-π stacking derived from tyrosine Y152 (TM3) and phenylalanine F323 (TM6) from the opposite side of the binding pocket. Aromatic moieties are surrounded by hydrophobic residues which are responsible for recognition of these ligands by hNET like the mentioned valine V148. The presented results constitute an extension of the published data [23,34] as we focused on many aspects of hNET, including the analysis of greater amount of inhibitors.

## 3. Materials and Methods

### 3.1. Sequences and Homology Models

For the study, the amino acid sequence of the human norepinephrine transporter (UniProt ID P23975) was downloaded. As templates to construct all the homology models, we used X-ray crystal structures of dopamine transporters from *Drosophila melanogaster* PDB IDs: 4xpg [49] and 4m48 [50], with resolutions 3.21 Å and 2.96 Å, respectively. The three-dimensional hNET structures were built using the SWISS-MODEL server [51]. Generated 4xpg and 4m48-based hNET models contained two sodium and one chloride ions inherited from dDAT templates. The 4xpg hNET also included a cocaine analog located at the binding pocket in X-ray 4xpg dDAT. All the presented models were selected according to knowledge-based QMEAN and GMQE values [33]. For multiple sequence alignment hNET (UniProt ID P23975), hDAT (UniProt ID Q01959), and hSERT (UniProt ID P31645) sequences were used. The process was carried out on the PROMALS3D multiple sequence and structure alignment server [27].

### 3.2. Ligands Preparation

All of the presented inhibitors were selected according to affinity and selectivity determined experimentally toward hNET and *Rattus norvegicus* rNET, taken from the ChEMBL database of bioactive molecules [42]. Ligands were prepared in the MAESTRO program using the Schrödinger LigPrep calculation package. The procedure involved taking into account ionization occurring at physiological pH (7.4 ± 0.2) with an availed OPLS_2005 (Optimized Potentials for Liquid Simulations) force field. Absolute configuration was determined for all the asymmetric carbon atoms. To obtain reliable results, the appropriate stereoisomers corresponding to those used with registered/inspected drugs were selected.

### 3.3. Docking

The first step involved default preparation of models in the Protein Preparation Wizard. For 4xpg hNET (x = 40.267, y = −1.411, z = −25.393) and 4m48 hNET (−40.899, 1.143, 57.732) grid centers were adopted with an inner box size of x = 22 Å, y = 22 Å, z = 22 Å, for both of them. The centers of binding sites were defined by residues F72, D75, Y151, Y152, F323, and D473 for 4xpg and 4m48 hNET models. The second step involved glide docking of selected compounds and was performed using a standard procedure with all the default settings. At both stages, an OPLS_2005 force field was applied.

## 4. Conclusions

To conclude, the constructed models of human norepinephrine transporter provide a good reference point for suggestions as to how the atomic structure of this protein might look. Meticulous examination of the spatial arrangement of initial elements engaged in ligand recognition from the entry pathway to high affinity binding sites allows for elucidation of the selectivity and placement of bioactive molecules. Models built on 4xpg and 4m48 dDAT templates possess a different spatial arrangement of hydrophobic phenylalanine F323 residues, which is important in catching aryl moieties from intracellular space. Several compounds may interact with residues not conserved, i.e., tyrosine Y151 and serine S423. Other findings showed the close relationship of the halogen atom with valine V148, experimentally proven as also being important in hNET ligand recognition. We hope that the presented data analysis may have an impact on understanding the molecular interactions between norepinephrine transporter and its inhibitors and have an impact on future development of novel bioactive compounds.

## Figures and Tables

**Table 1:** Summary of residues involved in ion binding represented for LeuT, monoamine transporters, and hNET 4xpg and 4m48 models. Primary differences in properties in amino acid residues are presented in bold letters.

Ion	aLeuT (2qju)	hDAT [23]	dDAT (4m48)	hNET [23]	hNET Models 4xpg and 4m48
Na^+^1	**leucine** (substrate)	**Asp79**	**Asp46** via **H_2_O**	**Asp75**	**Asp75**
	Ala22	Ala77	Ala44	Ala73	Ala73
	Asn27	Asn82	Asn49	Asn78	Asn78
	Thr254	Ser321	Ser320	Ser318	Ser318
	Asn286	Asn353	Asn352	Asn350	Asn350
Na^+^2	Gly20	Gly75	Gly42	Gly71	Gly71
	Val23	Val78	Val45	Val74	Val74
	Ala351	Leu418	Leu417	Leu415	Leu415
	**Thr354**	**Asp421**	**Asp420**	**Asp418**	**Asp418**
	Ser355	Ser422	Ser421	Ser419	Ser419
Cl^−^	**Lys121**	**Tyr102**	**Tyr69**	**Tyr98**	**Tyr98**
	Ser150	Ser321	Ser320	Ser318	Ser318
	Tyr151	Asn353	Asn352	Asn350	Asn350
	Ser165	Gln317	Gln316	Gln314	Gln314
	Phe167	Ser357	Ser356	Ser354	Ser354

**Table 2:** Docking GlideScore of selected compounds for hNET models on dDAT templates 4xpg and 4m48.

Compound	hNET Model 4xpg	hNET Model 4m48
Desipramine	−7.602	**−9.225**
Nortriptyline	−7.137	**−9.432**
Reboxetine	−7.071	**−8.158**
Bupropion	−6.584	**−6.859**
Duloxetine	**−7.859**	−7.295
Mazindol	**−8.845**	−8.624
Modafinil	−7.793	**−8.177**
Nocaine	**−7.392**	−7.269
Venlafaxine	−7.603	**−7.674**
Compound X	**−8.530**	−7.620

The values in bold indicate the model which resulted in a better binding mode of ligand and provided more beneficial value of scoring function.

## Abbreviations

5-HT	5-hydroxytryptamine, serotonin
ADHD	attention deficit hyperactivity disorder
BAT	Biogenic amine transporter
cDNA	Complementary DNA
CNS	Central nervous system
DAT	Dopamine transporter
dDAT	Drosophila dopamine transporter
EL2	Extracellular loop 2
EL4	Extracellular loop 4
GMQE	Global Model Quality Estimation
hDAT	Human dopamine transporter
hNET	Human norepinephrine transporter
IL1	Intracellular loop 1
LeuBAT	Leucine biogenic amine transporter
LeuT	Leucine transporter
MAT	Monoamine transporter
NDRIs	Norepinephrine/dopamine re-uptake inhibitors
NE	Norepinephrine
NET	Norepinephrine transporter
NRIs	Norepinephrine re-uptake inhibitors
NSSs	Neurotransmitter sodium symporters
PDB	Protein Data Bank
pK*i*	Negative logarithm of the dissociation constant of ligand—transporter complex
QMEAN	Qualitative Model Energy Analysis
SERT	Serotonin transporter
SLC6	Solute carrier family 6
SNRIs	Serotonin/norepinephrine re-uptake inhibitors
SSRIs	Selective serotonin re-uptake inhibitors
TCAs	Tricyclic antidepressants
TM	Transmembrane domain
